# Effect of Frozen Embryo Transfer and Progestin-primed Ovary Stimulation on IVF outcomes in women with high body mass index

**DOI:** 10.1038/s41598-017-07773-w

**Published:** 2017-08-07

**Authors:** Li Wang, Mingru Yin, Yali Liu, Qiuju Chen, Yun Wang, Ai Ai, Yonglun Fu, Zhiguang Yan, Wei Jin, Hui Long, Qifeng Lyu, Yanping Kuang

**Affiliations:** The department of Assisted Reproduction, Shanghai Ninth People’s Hospital, Shanghai Jiaotong University School of Medicine, Shanghai, 200011 People’s Republic of China

## Abstract

Among women undergoing IVF, high BMI negatively affects pregnancy outcomes when using the conventional ovary stimulating protocols combined with fresh embryo transfer. Therefore, finding a proper treatment for these high BMI women is more important and urgent when obesity is prevalent. In our study, we reported a retrospective study of 4457 women who were divided into normal BMI group (18.5 kg/m^2^–24.9 kg/m^2^) and high BMI group (≥25 kg/m^2^) undergoing 4611 IVF treatment cycles with frozen embryo transfer (FET). We found the high BMI group originally had the poor oocytes performance, but after FET they got the similar pregnancy outcomes as the normal BMI group. Then under FET we analyzed the IVF outcomes of our new progestin-primed ovary stimulation (PPOS) protocol, indicating that the hMG + MPA (4 or 10 mg/d) groups had the obvious better pregnancy results than the conventional short group in the high BMI group, and binary logistic regression analysis showed the hMG + MPA (10 mg/d) group was associated with better pregnancy outcomes than the hMG + MPA (4 mg/d) group. These results indicate PPOS - hMG + MPA (10 mg/d) combined with FET might be a new potential treatment choice for the high BMI women undergoing IVF treatments.

## Introduction

Obesity has been a worldwide health problem both in developed and developing countries, which is no effective strategy to prohibit its prevalence. According to the World Health Organization (WHO), if the body mass index (BMI) is equal to or greater than 25 kg/m^2^, it is considered to be overweight, whereas if it is equal to or greater than 30 kg/m^2^, it is considered obesity^1^. Over the past decade population-based trends show a 40% increase in pre-pregnancy overweight or obesity and a twofold increase in pre-pregnancy morbid obesity in women^2^. For the human reproductive system, high BMI negatively influences the fertility from endocrine disorders^3^, anovulation^4^ and decreasing in oocyte quality^5^, to endometrial receptivity which could induce the spontaneous abortion rate^6^. Therefore, more and more high BMI reproductive-age women have to fall back on assisted reproductive technology (ART) to resolve their infertility.

High BMI women undergoing ART also have lower chances of pregnancy and obstetric outcomes than non-obese women^7–9^. Women with a BMI more than 25 kg/m^2^ shows longer follicular phases, shorter luteal phases, and lower follicle-stimulating hormone (FSH), luteinizing hormone (LH), and progesterone metabolites^8^. In addition to this, the insulin resistance and leptin levels are increased and hyperandrogenemia occurs in obese women^6^. Debates about whether the oocyte quality or the endometrium mainly damages the fertility of obesity women exist several decades. Therefore, a systematic review and meta-analysis estimating the associations between BMI and IVF outcomes in donor oocyte recipients shows that high BMI does not affect IVF outcomes of obese women using donor oocytes, which indicates oocyte quality rather than endometrial receptivity may be the most important factor^10^. Different ovary stimulation protocols could get different quality oocytes, such as mild ovarian stimulation might yield healthier oocytes in obese women than conventional ovarian stimulation^5^, so seeking one ovary stimulation protocol which is adaptive for the high BMI women and could improve their oocyte quality is very urgent and important, except for encouraging them to lose weight before trying assisted ART.

However, using fresh embryo transfer (ET) may let the endometrial disturbance which is caused by ovary stimulation persist and influence ensuing pregnancy and obstetric outcomes in the ART women. And observational studies have shown that the FET group had significantly greater clinical pregnancy rate per transfer than the ET group^11^. Freezing technique appeared in 1996^12^, then the first birth using oocyte vitrification was reported in 1999^13^, around 10 years later the first pregnancy resulting from embryo vitrification came out^14^. As an important breakthrough in the reproductive biology, freezing technique was used wide spread throughout the world from 2004. So far, the oocyte and embryo cryopreservation could achieve at an excellent survival rate of over 90% by using it, furthermore pregnancy chances are equivalent to those with fresh transfers^15^ or sometimes even better^16^. Frozen-embryo transfer not only allows the ovary and exposed endometrial to recover from the ovarian stimulation, but also avoids the adverse effect of high estradiol levels on the implantation. Therefore, FET technology might heighten the efficiency of ART in the obese women and become the first choice.

Thanks for FET technology, a new ovary stimulation protocol using progesterone plus hMG appeared recently^17–22^. There are two ways of using progesterone, one is endogenous, as with luteal stimulation, another is exogenous, as with the use of progesterone in the follicular phase (progestin primed ovarian stimulation (PPOS))^23^. Kuang’s team firstly found Luteal-phase ovarian stimulation is feasible for producing competent oocytes/embryos in women undergoing IVF/ICSI treatments, with optimal pregnancy and obstetric outcomes in FET cycles. The luteal-phase ovarian-stimulation (LPS) protocol achieved an obvious higher implantation rate, pregnancy rate, live birth and ongoing pregnancy rate compared with the short-term protocol^22^. The Medroxyprogesterone acetate (MPA) group had a similar clinical pregnancy rates, implantation rates, and live-birth rates compared with the short-term protocol. But for the polycystic ovary syndrome (PCOS) patients, progesterone could significantly improve their pregnancy and live birth outcomes^24, 25^. All these results indicate progesterone is a new feasible ovary stimulation protocol, even may improve the oocyte developmental potential and enhance the final clinical pregnancy and live birth outcomes in the special IVF group, such as PCOS patients.

The high BMI women have some similar characters as the PCOS women, such as lower progesterone level. Whether PPOS protocol combined with FET might become an adaptive therapy for the high BMI women, which seems very hopeful and meaningful. Especially look around the current therapies for the high BMI women, most are the standard long GnRH agonist protocol, the micro-dose GnRH flare protocol, the GnRH antagonist protocol, the over-length protocol, or the improved over-length protocol^26–29^ combined with ET, which always get decreased pregnancy and live birth outcomes. Therefore, we do this retrospective analysis to research the oocyte and pregnancy outcomes of PPOS combined with FET treatment strategy in the high BMI women.

## Results

### Clinical characteristics and IVF outcomes in the two BMI groups under the FET technology

**Figure 1:**
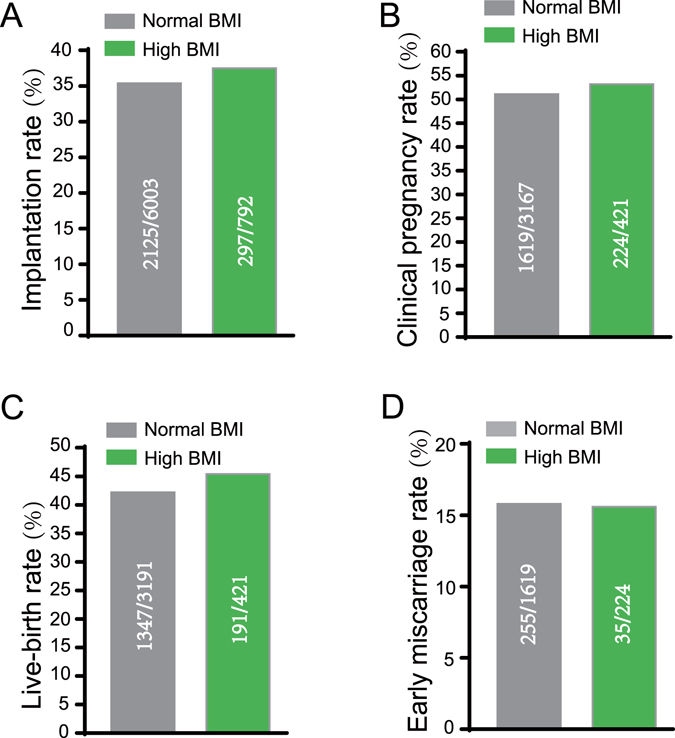
The normal BMI and high BMI groups did not have difference in the implantation rate (**A**), clinical pregnancy rate (**B**), live-birth rate (**C**) and early miscarriage rate (**D**) after FET. The raw data were shown in the figures and analyzed by Chi-square test. The differences were considered statistically significant when the *p-value* was less than 0.05.

Table 1 shows the baseline characteristics and IVF outcomes by using FET technology in the patients of two BMI groups. There were 2212 normal BMI women and 320 high BMI women (21.28 vs. 27.13; *p* < 0.001) enrolled. There were no differences in the age, duration of infertility, and numbers of antral follicles. Consistent with the previous research^26–29^, the basal FSH (5.00 vs. 5.66, *p* < 0.001), E2 (28.90 vs. 33.85, *p* < 0.001), P (0.22 vs. 0.29, *p* < 0.001) level in the high BMI group were lower than that in the normal BMI group. Although the mean ovarian hMG stimulation duration (9.4 days vs. 8.6 days, *p* < 0.001) and hMG dose (2008 vs. 1807, *p* < 0.001) of the high BMI group was significantly higher than that in the normal BMI groups, the oocyte performance of high BMI women was not satisfactory. The high BMI group had the lower numbers of oocytes retrieved (10.98 vs. 12.53, *p* < 0.001), MII oocytes (6.20 vs. 7.25, *p* < 0.001), fertilized oocytes (6.10 vs. 7.02, *p* < 0.01), Top-quality embryos (3.27 vs. 3.65, *p* < 0.05) and viable embryos (3.64 vs. 4.08, *p* < 0.01) when compared with the normal BMI group. Unexpectedly, after FET the high BMI group had the similar implantation (37.5% vs. 35.4%), clinical pregnancy rate per transfer (53.2% vs. 51.1%), live-birth rate per transfer (45.4% vs. 42.2%) and early miscarriage rate (15.6% vs. 15.8%) comparing with the normal BMI group in the Fig. 1. In our results, the high BMI group had poor outcomes of oocyte performance than the normal BMI group as previous research reported, including the oocytes retrieved, MII oocytes, fertilized oocytes, top-quality embryos and viable embryos. Interestingly, this adverse tendency in the oocyte performance was changed after using FET, and the high BMI group got the similar clinical outcomes as the normal BMI group. As we know, FET permits the longer endometrium recovery time from the disturbance caused by ovary stimulation compared with ET. These unexpected and satisfactory IVF clinical outcomes indicated that FET might be a preferential choice for these high BMI women.

**Table 1:** Clinical characteristics and oocytes outcomes under the frozen-thawed embryo transfer technology according to female BMIs. Student’s *t*-tests or Mann-Whitney *U*-test were applied. Data are presented as mean ± standard error of the mean. The differences were considered statistically significant when the *p-value* was less than 0.05.

Parameter	Normal BMI	High BMI	*P*
	18.5–24.99	≥25	
Patiens (n)	2212	320	
Age (y)	31.25 ± 0.08	31.57 ± 0.208	0.200
BMI(kg/m^2^)	21.28 ± 0.04	27.13 ± 0.14	<0.001
Duration of infertility (y)	3.27 ± 0.05	3.51 ± 0.15	0.320
Day3 FSH (IU/L)	5.66 ± 0.03	5.00 ± 0.10	<0.001
Day3 E2 (pg/mL)	33.85 ± 0.29	28.90 ± 0.88	<0.001
Day3 P (ng/mL)	0.29 ± 0.01	0.22 ± 0.01	<0.001
Day3 AFC	11.20 ± 0.09	11.68 ± 0.27	0.150
hMG duration (d)	8.62 ± 0.04	9.44 ± 0.13	<0.001
hMG dose (IU)	1807 ± 9.59	2008 ± 40.05	<0.001
Oocytes retrieved (n)	12.53 ± 0.16	10.98 ± 0.41	<0.001
MII oocytes (n)	7.25 ± 0.12	6.20 ± 0.31	<0.001
Fertilized oocytes (n)	7.02 ± 0.11	6.10 ± 0.30	0.004
Top-quality embryos (n)	3.65 ± 0.07	3.27 ± 0.18	0.018
Viable embryos	4.08 ± 0.07	3.64 ± 0.18	0.004

### Cycle characteristics and oocytes outcomes in three ovarian stimulation groups according to female BMIs

Combined with the FET technology, we then analyzed the IVF outcomes of our three different ovary stimulation groups: conventional short, hMG + MPA (4 mg/d), and hMG + MPA (10 mg/d) separately in the normal BMI (18.5–24.9 kg/m^2^) and high BMI (≥25 kg/m^2^) women groups. Baseline characteristics, hormonal profiles and cycle characteristics of patients were presented in Table 2. Firstly, we analyzed the IVF outcomes of the same BMI women using three different ovary stimulation groups in Table 2. For the normal BMI women group, the duration of hMG was significantly longer in the hMG + MPA (10 mg/d) group than that in conventional short and hMG + MPA (4 mg/d) group (8.94 vs. 8.14 vs. 6.87, *p* < 0.05 for any two groups); the dose of hMG in the hMG + MPA (4 mg/d) group was more than that in the hMG + MPA (10 mg/d) and conventional short groups (2002 vs. 1939 vs. 1583; *p* < 0.05 for any two groups); the numbers of oocytes retrieved were not significantly different across all three groups; the MII oocytes were similar in the hMG + MPA (4 or 10 mg/d) groups (8.82 vs. 8.62), but both significantly more than that in the conventional short group (8.82 vs. 7.68, *p* < 0.05; 8.62 vs. 7.68, *p* < 0.05); the fertilized oocytes in the hMG + MPA (10 mg/d) group were similar as conventional short group (7.68 vs. 7.36), but significantly more than hMG + MPA (4 mg/d) group (7.68 vs. 7.08, *p* < 0.05); the top-quality embryos (3.68 vs. 3.77) were similar in the hMG + MPA (10 mg/d) and conventional short groups, but both significantly more than that in hMG + MPA (4 mg/d) group (3.68 vs. 2.95, p < 0.05; 3.77 vs. 2.95, *p* < 0.05); the viable embryos in the conventional short group were similar as hMG + MPA (10 mg/d) group (4.40 vs. 4.24), but significantly more than hMG + MPA (4 mg/d) group (4.40 vs. 3.97, *p* < 0.05). For the high BMI women group, the hMG duration were similar in the conventional short and hMG + MPA (10 mg/d) groups (9.48 vs. 9.44), but both significantly longer than that in the hMG + MPA (4 mg/d) group (9.48 vs. 7.05, *p* < 0.05; 9.44 vs 7.05, *p* < 0.05); The hMG dose in the hMG + MPA (4 mg/d) group was similar as hMG + MPA (10 mg/d), but significantly more than conventional short groups (2171 vs. 1962, *p* < 0.05); the MII oocytes was similar in the hMG + MPA (4 or 10 mg/d) groups (8.03 vs. 7.68), but both significantly more than that in the conventional short group (8.03 vs. 5.78, *p* < 0.05; 7.68 vs 5.78, *p* < 0.05); the oocytes retrieved, the fertilized oocytes, the top-quality and viable embryos were similar among the three ovary stimulation groups.

**Table 2:** Cycle’s characteristics and oocytes outcomes in the three ovarian stimulation groups according to female BMIs. One-way ANOVA analysis, the nonparametric test, student’s *t*-tests or Mann-Whitney *U*-test were applied. Data are presented as mean ± standard error of the mean. The differences were considered statistically significant when the *p-value* was less than 0.05. When compared with the *p-value* of subgroups in the same BMI group, the different superscript alphabets of a, b, c stand for a significant difference (*p* < 0.05). When compared with the *p-value* of the normal BMI group and high BMI group in the same ovary stimulation group, the symbols *, ^#^, ^※^ separately stands for a significant difference (*p* < 0.05) in the conventional short, HMG + MPA (4 mg) and HMG + MPA (10 mg) ovary stimulation group.

	Normal BMI (18.5–24.99)				High BMI (≥25)			
	Conventional short (n = 1190)	HMG + MPA (4 mg) (n = 1331)	HMG + MPA (10 mg) (n = 1412)	*P*	Conventional short (n = 232)	HMG + MPA (4 mg) (n = 240)	HMG + MPA (10 mg) (n = 208)	*P*
Patients (n)	1160	1272	1383		217	226	199	
Age (y)	31.29 ± 0.10^a^	31.53 ± 0.10^a^	31.43 ± 0.10^a^	0.222	31.67 ± 0.23^a^	32.19 ± 0.26^a,#^	31.99 ± 0.26^a,※^	0.315
BMI (kg/m^2^)	21.30 ± 0.05^a^	21.35 ± 0.05^a^	21.24 ± 0.04^a^	0.230	27.20 ± 0.15^a,*^	27.38 ± 0.23^a,#^	27.29 ± 0.19^a,※^	0.787
Duration of infertility (y)	3.28 ± 0.06^a^	3.05 ± 0.07^b^	3.18 ± 0.07^a,b^	0.052	3.82 ± 0.16^a,*^	3.39 ± 0.19^a^	3.37 ± 0.21^a^	0.167
Indication, n (%)	^a^	^a^	^a^	0.447	^a^	^a^	^a^	0.466
Tubal factor	772 (64.9)	817 (61.4)	873 (61.8)		140 (60.3)	147 (61.3)	121 (58.7)	
Male factor	91 (7.6)	91 (6.8)	117 (8.3)		26 (11.2)	20 (8.3)	13 (6.3)	
Unexplained factor	53 (4.5)	80 (6.0)	69 (4.9)		10 (4.3)	12 (5.0)	15 (7.3)	
Combination of factors	274 (23.0)	343 (25.7)	353 (25.0)		56 (24.1)	61 (25.4)	57 (27.7)	
Day3 FSH (IU/L)	5.70 ± 0.03^a^	5.80 ± 0.04^a^	5.80 ± 0.04^a^	0.084	5.40 ± 0.09^a,*^	5.62 ± 0.08^a,#^	5.69 ± 0.10^a^	0.063
Day3 E2 (pg/mL)	34.12 ± 0.39^a^	35.26 ± 0.36^a^	34.89 ± 0.40^a^	0.116	30.24 ± 0.89^a,*^	32.18 ± 0.81^a,#^	32.39 ± 1.04^a,※^	0.192
Day3 P (ng/mL)	0.29 ± 0.01^a^	0.31 ± 0.01^a^	0.31 ± 0.01^a^	0.131	0.26 ± 0.02^a^	0.25 ± 0.01^a,#^	0.27 ± 0.02^a^	0.223
Day3 AFC	11.02 ± 0.12^a^	11.10 ± 0.12^a^	11.07 ± 0.13^a^	0.888	11.02 ± 0.31^a^	11.23 ± 0.31^a^	11.73 ± 0.38^a^	0.311
hMG duration (d)	8.14 ± 0.05^a^	6.87 ± 0.16^b^	8.94 ± 0.04^c^	<0.001	9.48 ± 0.19^a,*^	7.05 ± 0.27^b^	9.44 ± 0.17^a,※^	<0.001
hMG dose (IU)	1583 ± 14.09^a^	2002 ± 8.60^b^	1939 ± 11.30^c^	<0.001	1962 ± 59.02^a,*^	2171 ± 30.58^b,#^	2082 ± 49.26^a,b,※^	0.005
Oocytes retrieved (n)	11.03 ± 0.19^a^	10.71 ± 0.18^a^	11.03 ± 0.16^a^	0.360	8.55 ± 0.44^a,*^	9.41 ± 0.39^a,#^	9.96 ± 0.48^a,※^	0.075
MII oocytes (n)	7.68 ± 0.14^a^	8.82 ± 0.16^b^	8.62 ± 0.15^b^	<0.001	5.78 ± 0.32^a,*^	8.03 ± 0.36^b^	7.68 ± 0.42^b※^	<0.001
Fertilized oocytes (n)	7.36 ± 0.14^a,b^	7.08 ± 0.14^a^	7.68 ± 0.14^b^	0.008	5.59 ± 0.31^a,*^	6.40 ± 0.31^a^	6.59 ± 0.39^a,※^	0.083
Top-quality embryos (n)	3.77 ± 0.09^a^	2.95 ± 0.09^b^	3.68 ± 0.09^a^	<0.001	2.81 ± 0.19^a,*^	2.62 ± 0.18^a^	3.17 ± 0.23^a,※^	0.153
Viable embryos (n)	4.40 ± 0.09^a^	3.97 ± 0.09^b^	4.24 ± 0.09^a,b^	0.002	3.38 ± 0.20^a,*^	3.59 ± 0.21^a^	3.68 ± 0.22^a,※^	0.592

Furthermore, we analyzed the IVF outcomes of two BMI women groups when using the same ovarian stimulation treatment in Table 2. For the conventional short treatment, the high BMI women group has significantly lower basal FSH (5.40 vs. 5.70, *p* < 0.01) and E2 level (30.24 vs. 34.12, *p* < 0.01), higher ovarian hMG stimulation duration (9.48 vs. 8.14, *p* < 0.01) and hMG dose (1962 vs. 1583, *p* < 0.01), but less oocytes retrieved (8.55 vs. 11.03, *p* < 0.01), MII oocytes (5.78 vs. 7.68, *p* < 0.01), fertilized oocytes (5.59 vs. 7.36, *p* < 0.01), top-quality embryos (2.81 vs. 3.77, *p* < 0.01) and viable embryos (3.38 vs. 4.40, *p* < 0.01) than the normal BMI women group. For the hMG + MPA (4 mg/d) treatment, the high BMI women group also had significantly lower basal FSH (5.62 vs. 5.80, *p* < 0.05), E2 (32.18 vs. 35.26, *p* < 0.01) and P level (0.25 vs. 0.31, *p* < 0.01), higher ovarian hMG stimulation dose (2171 vs. 2002, *p* < 0.01), but less oocytes retrieved (9.41 vs. 10.71, *p* < 0.01), similar MII oocytes (8.03 vs. 8.82), fertilized oocytes (6.40 vs. 7.08), top-quality embryos (2.62 vs. 2.95) and viable embryos (3.59 vs. 3.97) compared with the normal BMI women group. For the hMG + MPA (10 mg/d) treatment, the high BMI women group had significantly lower basal E2 (32.39 vs. 34.89, *p* < 0.05), similar FSH and P level, higher ovarian hMG stimulation duration (9.44 vs. 8.94, *p* < 0.01) and hMG dose (2082 vs. 1939, *p* < 0.01), less oocytes retrieved (9.96 vs. 11.03, *p* < 0.05), MII oocytes (7.68 vs. 8.62, *p* < 0.05), fertilized oocytes (6.59 vs. 7.68, *p* < 0.01), top-quality embryos (3.17 vs. 3.68, *p* < 0.05) and viable embryos (3.68 vs. 4.24, *p* < 0.05) than the normal BMI women group.

### Pregnancy outcomes in FET cycles from three ovarian stimulation groups according to female BMIs

**Figure 2:**
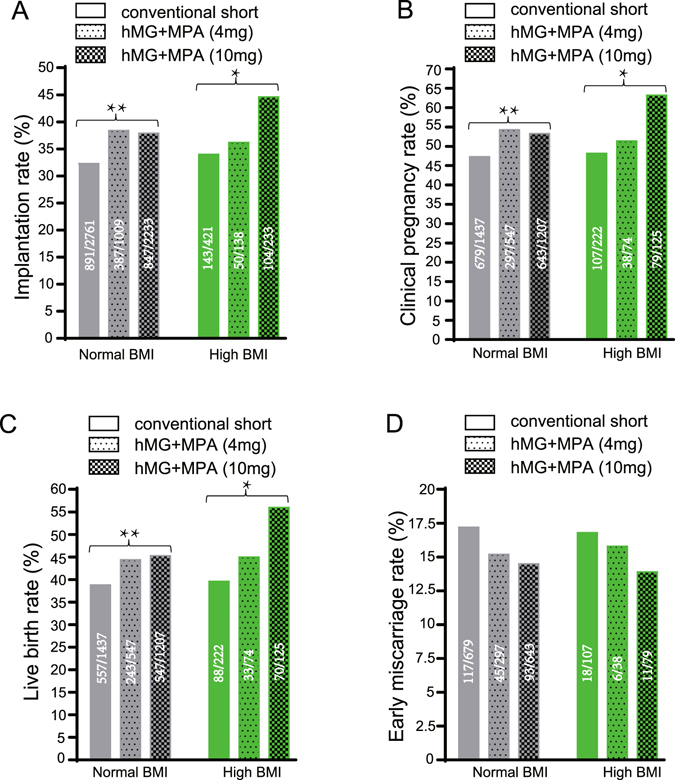
Comparison of implantation rate (**A**), clinical pregnancy rate (**B**), live-birth rate (**C**) and early miscarriage rate (**D**) across the three ovary stimulation groups according to female BMIs under FET. The raw data were shown in the figures and analyzed by Chi-square test. *p < 0.05, **p < 0.01. Data are presented as mean ± standard error of the mean.

Table 3 describes the FET characteristics of three ovarian stimulation groups separately in the two different BMI women groups. Similarly, we first analyze the pregnancy and live birth outcomes of the same BMI women using three different ovary stimulation treatments in Fig. 2. For the normal BMI women group, the biochemical pregnancy rate (56.9 vs. 58.7), implantation rate (38.4 vs. 37.9), clinical pregnancy rate (54.3 vs. 53.3) and live-birth rate (44.4 vs. 45.3) were similar in the hMG + MPA (4 or 10 mg/d) groups, but significantly higher than that in the conventional short group (*p* < 0.05 for any two groups). For the high BMI women group, the hMG + MPA (10 mg/d) group had significantly higher biochemical pregnancy rate (70.4 vs. 52.7 vs. 53.2, *p* < 0.01), the implantation rate (44.6 vs. 36.2 vs. 34.0, *p* < 0.05), clinical pregnancy rate (63.2 vs. 51.4 vs. 48.2, *p* < 0.05) and live-birth rate (56.0 vs. 45.0 vs. 39.6, *p* < 0.05) than that in the hMG + MPA (4 mg/d) and short groups. The early miscarriage rate was not significantly different among all three treatment groups in the normal and high BMI women.

**Table 3:** Women and cycle’s characteristics of frozen-thawed embryos originating from three ovarian stimulation groups according to female BMIs. One-way ANOVA analysis, the nonparametric test, student’s *t-*tests or Mann-Whitney *U*-test were applied. Data are presented as mean ± standard error of the mean. The differences were considered statistically significant when the *p-*value was less than 0.05. When compared with the *p*-value of subgroups in the same BMI group, the different superscript alphabets of a, b, c stand for a significant difference (*p* < 0.05). When compared with the *p*-value of the normal BMI group and high BMI group in the same ovary stimulation group, the symbols *, ^#^, ^※^ separately stands for a significant difference (*p* < 0.05) in the conventional short, HMG + MPA (4 mg) and HMG + MPA (10 mg) ovary stimulation group.

	Normal BMI (18.5–24.99)				High BMI (≥25)			
	Conventional short	HMG + MPA (4 mg)	HMG + MPA (10 mg)	*P*	Conventional short	HMG + MPA (4 mg)	HMG + MPA (10 mg)	*P*
Patients (n)	947	413	852		162	58	100	
Age (y)	31.31 ± 0.12^a^	31.16 ± 0.17^a^	31.24 ± 0.12^a^	0.762	31.71 ± 0.29^a^	32.02 ± 0.55^a^	31.56 ± 0.38^a^	0.389
BMI (kg/m^2^)	21.28 ± 0.06^a^	21.42 ± 0.08^a^	21.22 ± 0.06^a^	0.141	27.20 ± 0.19^a,*^	26.99 ± 0.23^a,#^	27.11 ± 0.29^a,※^	0.858
Duration of infertility (y)	3.32 ± 0.07^a^	3.25 ± 0.12^a^	3.21 ± 0.08^a^	0.585	3.69 ± 0.19^a,*^	3.59 ± 0.41^a^	3.19 ± 0.27^a^	0.333
FET cycles (n)	1437	547	1207		222	74	125	
Thawed embryos (n)	2784	1012	2252		425	139	234	
Viable embryos after thawed (n)	2761	1009	2233		421	138	233	
Transferred embryos (n)	1.92 ± 0.01^a^	1.85 ± 0.02^b^	1.85 ± 0.01^b^	<0.001	1.90 ± 0.02^a^	1.87 ± 0.04^a^	1.86 ± 0.03^a^	0.648
Endometrial thickness (mm)	10.87 ± 0.07^a^	10.93 ± 0.13^a^	11.00 ± 0.07^a^	0.374	10.74 ± 0.15^a^	11.19 ± 0.37^a^	11.20 ± 0.20^b^	0.132
Endometrial preparation of the recipients, n (%)	^a^	^b^	^a,b^	0.030	^a^	^a^	^a,※^	0.628
Natural cycle	427 (29.7)	130 (23.8)	331 (27.4)		53 (23.9)	17 (23.0)	31 (24.8)	
Hormone replacement	499 (34.7)	224 (41.0)	466 (38.6)		82 (36.9)	30 (40.5)	38 (30.4)	
Letrozole or late stimulation	511 (35.6)	193 (35.3)	410 (34.0)		87 (39.2)	27 (36.5)	56 (44.8)

### Logistic regression of three ovarian stimulation treatment groups on the pregnancy outcomes in the two BMI groups

Table 4 shows the binary logistic regression results of pregnancy outcomes separately in the same BMI women using three different ovarian stimulation treatments combined with FET. After adjusting for female age, duration of infertility, basic serum FSH level, the E2, P level and endometrial thickness on the FET day and the number of embryos transferred, our study showed that for the normal BMI women, compared with the conventional short group (ref.), the hMG + MPA (4 mg/d) and hMG + MPA (10 mg/d) groups had similar but significantly higher odds of implantation rate (hMG + MPA (4 mg/d) group: OR 1.42, 95% CI 1.13–1.78, *p* < 0.01; hMG + MPA (10 mg/d) group: OR 1.37, 95% CI 1.15–1.62, *p* < 0.01), clinical pregnancy rate (hMG + MPA (4 mg/d) group: OR 1.51, 95% CI 1.21–1.90, *p* < 0.01; hMG + MPA (10 mg/d) group: OR 1.40, 95% CI 1.18–1.65, *p* < 0.01), and live birth rate (hMG + MPA (4 mg/d) group: OR 1.47, 95% CI 1.17–1.84, *p* < 0.01; hMG + MPA (10 mg/d) group: OR 1.39, 95% CI 1.17–1.65, *p* < 0.01). Statistical significance of outcomes was confirmed with the use of ANOVA. For the high BMI women, compared with the conventional short group (ref.), the hMG + MPA (10 mg/d) group had significantly higher odds of implantation rate (OR 1.92, 95% CI 1.19–3.11, *p* < 0.01), clinical pregnancy rate (OR 1.92, 95% CI 1.19–3.11, *p* < 0.01), and live birth rate (OR 1.82, 95% CI 1.13–2.94, *p* < 0.01), than the hMG + MPA (4 mg/d) group (implantation rate: OR 1.26, 95% CI 0.70–2.28; clinical pregnancy rate: OR 1.32, 95% CI 0.73–2.37; live birth rate: OR 1.30, 95% CI 0.72–2.36). No significant difference was found in the early miscarriage rate among all three ovarian stimulation groups in the normal and high BMI women.

**Table 4:** Logistic regression of three ovarian stimulation groups combined with FET on the pregnancy outcomes according to female BMIs. After adjusting for female age, duration of infertility, basic serum FSH level, the E2, P level and endometrial thickness on the FET day, and the number of embryos transferred, binary logistic regression was performed. The differences were considered statistically significant when the *p-value* was less than 0.05.

	Normal BMI (18.5–24.99)			High BMI (≥25)		
	Conventional short	HMG + MPA (4 mg)	HMG + MPA (10 mg)	Conventional short	HMG + MPA (4 mg)	HMG + MPA (10 mg)
Implanation rate, %	32.3	38.4	37.9	34.0	36.2	44.6
aOR (95% CI)	Reference	1.42 (1.13–1.78)	1.37 (1.15–1.62)	Reference	1.26 (0.70–2.28)	1.92 (1.19–3.11)
*P* value		0.002	0.000		0.434	0.008
Clinical pregnancy rate, %	47.3	54.3	53.3	48.2	51.4	63.2
aOR (95% CI)	Reference	1.51 (1.21–1.90)	1.40 (1.18–1.65)	Reference	1.32 (0.73–2.37)	1.92 (1.19–3.11)
*P* value		0.000	0.000		0.359	0.008
Early miscarriage rate, %	17.2	15.2	14.5	16.8	15.8	13.9
aOR (95% CI)	Reference	0.92 (0.61–1.40)	0.94 (0.69–1.28)	Reference	0.91 (0.28–2.98)	1.42 (0.58–3.53)
*P* value		0.704	0.675		0.871	0.445
Live birth rate, %	38.8	44.4	45.3	39.6	45.0	56.0
aOR (95% CI)	Reference	1.47 (1.17–1.84)	1.39 (1.17–1.65)	Reference	1.30 (0.72–2.36)	1.82 (1.13–2.94)
*P* value		0.001	0.000		0.390	0.014

## Discussion

Our IVF outcomes indicate that under control standard short term protocol or our initially used PPOS protocol (4 or 10 mg/day), the high BMI women group got the poorer oocyte performance than the normal BMI women group, but after FET this adverse situation in the oocyte performance of high BMI women was changed and achieved the similar pregnancy and live birth outcomes as the normal BMI women. Then within FET, we further analyzed the IVF outcomes of these three different ovary stimulation groups separately in the normal and high BMI women group. We found for the normal BMI women, the hMG + MPA (4 or 10 mg/d) groups got similar or significantly better oocytes, pregnancy and live birth results than the conventional short group, for the High BMI women the hMG + MPA (10 mg/d) group had the best oocytes, pregnancy and live birth outcomes in the three groups. Finally, we used the binary logistic regression to analyze the IVF outcomes of these three different ovary stimulation groups, and found compared with the conventional short protocol (ref), for the Normal BMI women the hMG + MPA (4 or 10 mg/d) groups had the similar and higher odds of pregnancy and live birth, but for the high BMI women the hMG + MPA (10 mg/d) group had the highest odds of pregnancy and live birth in three of them. These results indicate that combined with FET, the hMG + MPA (4 or 10 mg/d) ovarian stimulation treatment groups had the better pregnancy and live birth results than the conventional short group, regardless of in the normal or high BMI group. Moreover, the advantage in the pregnancy and live birth outcomes of hMG + MPA (10 mg/d) treatment is more obvious and prominent for the high BMI women, showing it may be a potential and more appropriate treatment for the high BMI women.

The obese women had lower pregnancy and live birth outcomes when ET was used in ART cycles, which was concluded from huge database derived from the multicenter trials^26, 27, 30^. Therefore, the debate between oocyte quality and endometrium receptivity, which is the main reason of that in the high BMI woman remains as before. However, high BMI does not affect IVF outcomes in obese women using donor oocytes, which indicates oocyte quality rather than endometrial receptivity may be the most important factor in obese women^10^. Our results showed that under three ovary stimulation treatments, the high BMI group (BMI ≥ 25) originally had the decreased retrieved oocytes, MII oocytes, fertilized oocytes, top-quality embryos and viable embryos than the normal BMI group, but after FET this adverse situation of oocyte performance in high BMI women was changed and achieved the similar pregnancy outcomes as the normal BMI women. Using FET might reduce the endometrium disturbance induced by the ovary simulation.

Observational studies have shown that the FET group had significantly greater clinical pregnancy rate per transfer than in the ET group. The results of women with the PCOS showed that FET was associated with a higher rate of live birth, a lower risk of the ovarian hyper-stimulation syndrome after the first transfer than fresh-embryo transfer. These results strongly showed that it impaired the endometrial receptivity in fresh ET cycles after ovarian stimulation, comparing to FET cycles with artificial endometrial preparation^11, 31^. FET not only allows the ovary and exposed endometrium to recover from the ovarian stimulation, but also avoids the adverse effect of high estradiol levels on the implantation. The increased risks of pregnancy loss^32^ have been associated with higher estradiol levels after IVF, which are much higher in fresh cycles than that in frozen cycles. Higher live-birth rates have been reported with the use of letrozole, which would result in lower mid luteal estrogen levels, as an ovulation stimulating agent than with clomiphene^33^. Thus it can be seen that the FET cycles could improve the endometrial status and shied the adverse impact of high estrogen levels inducing by the ovary stimulation, consequently had higher clinical pregnancy rate, live birth and lower pregnancy loss.

Then we further analyzed the IVF outcomes of these three different ovary stimulation groups with FET separately in the normal and high BMI women. We found that hMG + MPA (4 or 10 mg/d) treatment groups got the similar or significantly better oocytes, pregnancy and live birth results than the conventional short group in the normal BMI women; and for the high BMI women, the hMG + MPA (10 mg/d) group had the best oocytes, pregnancy and live birth outcomes in three of all. In spite of oral contraceptives mainly consisted of progesterone could inhibit gonadotropin secretion by negative feedback^34^ and may have a remnant adverse effect on the subsequent fresh embryo transfer cycle used for menses induction, which was associated with the lower level of LH, estradiol and decreased endometrium thickness^35^, but rates of pregnancy and live birth after FET were not affected by oral contraceptives used for scheduling endometrial preparation. Even a meta-analysis showed in GnRH agonist cycles that progestin pretreatment was associated with higher clinical pregnancy rate than placebo or no pretreatment^36^. Moreover, luteal-phase ovarian stimulation group had a significantly increased cumulative pregnancy rate per initiated stimulation cycle than mild treatment in patients with a normal ovarian reserve^22^, as well MPA group had slightly higher implantation and live-birth rate^17^. Especially for the PCOS patients who are in a progesterone deficiency state, the Utrogesten or Medroxyprogesterone Acetate with hMG protocols could significantly improve the pregnant outcomes, such as the biochemical pregnancy, clinical pregnancy rate and implantation rate^24, 25^. The high BMI women (BMI ≥ 25) also have the lower progesterone level^37, 38^ like the PCOS women, so the hMG + MPA (4 or 10 mg/d) protocol may just reverse this deficiency of progesterone and significantly improve pregnancy and live birth outcomes than the conventional short group. Moreover, the hMG + MPA (10 mg/d) group had better clinical pregnancy and live birth outcomes than the hMG + MPA (4 mg/d) group in the high BMI women group, not the normal BMI women group. That is may due to the hMG + MPA (10 mg/d) group could rescue the progesterone deficiency close to the physiological state in the high BMI women group compared with hMG + MPA (4 mg/d) group, which may not occur in the normal BMI women group. Futhermore, we should consider that the endometrial preparation is significantly different in the normal BMI group, which is might be a potential covariate to the pregnancy and live birth outcomes in the logistic regression analysis.

On the other hand, we consider that the hMG + MPA (4 or 10 mg/d) treatments may partly improve the oocyte developmental competence, which is defined as the ability of oocyte to compete meiosis, undergo fertilization, embryo genesis and term development^39^. Although the effects of progesterone on oocytes and embryo development have been assessed by some investigations, and always have the contradictory results both *in vitro* and *in vivo*. As a dominant steroid in follicular fluid at approximately 18 hours after the luteinizing hormone (LH) surge may have a critical role in maturation of oocytes at the germinal stage^40^, progesterone could influence the oocyte nuclear maturation, cytoplasmic maturation and development, in order to improve oocyte developmental competence^41^. Firstly progesterone plays an important role in the resumption of meiosis and progression to MII in oocytes, so it is strongly associated with oocyte quality and maturity^42^. And in rhesus monkeys, the improvement of *in vitro* oocyte development was demonstrated in the presence of progesterone and E2^43^. Secondly, progesterone could affect the relationship between cumulus cells and oocytes, which is important for oocyte cytoplasmic maturation and subsequent developmental competence^44^. It would appear that the progesterone, PGR and Ptgs2 are active participants in a positive loop of sustained EGFR induced-ERK signaling required of cumulus cell expansion and oocyte maturation during the ovulation process^45^. Thirdly, studies in periovulatory granulosa cells show P4 could protect the oocyte from the onset of apoptosis, mainly mediated by the classical PGR^46, 47^, and lower rates of apoptosis in cumulus cells have been correlated with developmental potential in both human and cattle oocytes^48–50^. In addition, progesterone could increase subsequent sperm head decondensation, male pronuclei formation^51^ and fertilization^52^ when it increased in pocine oocyte maturation medium. Finally, progesterone influences the cytoplasmic maturation of porcine oocytes, at least partially, by decreasing their polyadenylation, thereby altering maternal gene expression^53^.

There are also limitations of our study. First, this is a retrospective design of study, and need to be confirmed by randomized controlled trial in the future. Second, the high BMI group in our study just had one group (BMI ≥ 25), which is better when divided more accurately if have enough cycles, such as into overweight (BMI 25.0–29.0), obese (BMI 30.0–49.9) and superobese (BMI > 50). Whereas the two hMG + MPA groups both improved the clinical pregnant outcomes in this one high BMI group, and the more usage of MPA, the better IVF outcomes. These two hMG + MPA (4 or 10 mg/day) groups has the consistent and dose-dependent therapeutic effects in the high BMI group, which helps confirm the effect of MPA in the high BMI women. Thirdly, we did not have ET control in the high BMI group, which could help us to know the improvement of pregnancy outcomes in the high BMI women who originally had the poor oocyte performance than the normal BMI women is directly induced by FET or other factors. However, so many former researches show when using ET, the high BMI women would pursue this adverse performance in the pregnancy as in the oocyte.

In conclusion, it can be assumed that hMG + MPA (4 or 10 mg/d) ovary stimulation protocol combined with FET technology could be an efficient and acceptable treatment for high BMI (≥25) women comparing with the conventional short protocol, and for the high BMI women the hMG + MPA (10 mg/d) is the most effective treatment strategy in three of them. The results suggest that hMG + MPA (4 or 10 mg/d) protocol could be better effectiveness, with many theoretical advantages, such as FET could allow the ovary and exposed endometrial to be better recovered from the ovarian stimulation, avoiding of the adverse effect of high estradiol levels on the implantation, and MPA could partly improve the oocyte developmental competence, furthermore MPA (10 mg) greatly complement the deficiency of progesterone in the high BMI women and have higher probability in success of IVF.

## Methods

We performed a retrospective cohort study using the data from the Department of Assisted Reproduction of the Ninth People’s Hospital of Shanghai Jiaotong University School of Medicine database of 2006–2016. This study was approved by the Ethics Committee (Institutional Review Board) of the Ninth People’s Hospital of Shanghai. And the study was conducted according to the Declaration of Helsinki for medical research and informed consent was obtained. The characteristics and cycle parameters of the patients were recorded in the database of our center. We screened the database according to the following search criteria. Inclusion criteria included 1) age no older than 40; 2) basal serum FSH concentration of no more than 10 IU/L; 3) antral follicle count (AFC) of more than 3 on menstrual cycle day 2–3; and 4) regular menstrual cycles over the previous 3-month period (25–35 days in duration); and 5) women with infertility diagnoses of male, unexplained, and tubal factors. The exclusion criteria were as follows: 1) receipt of hormone treatments within the previous 3-month period; 2) diagnosis of polycystic ovarian syndrome; 3) presence of a functional ovarian cyst with E_2_ > 100 pg/mL; 4) preimplantation genetic diagnosis, *in vitro* maturation (IVM), and donor sperm were used; 5) any contraindications to ovarian stimulation treatment; 6) presence of fresh embryo transplantation.

### Body Mass Index Assessment

Weight and height were used to calculate BMI according to the standard formula: BMI = weight/height^2^ (kg/m^2^). Appropriate study subjects were divided into two groups depending on the BMI of patients and based on World Health Organization standards: normal BMI group (18.5 kg/m^2^ ≤ BMI < 25 kg/m^2^) and high BMI group (BMI ≥ 25 kg/m^2^)^1^. And BMI data was based on measured height and weight information in each patient’s medical record.

### Ovarian Stimulation Protocols

#### Conventional short ovarian stimulation protocol

A standard short term protocol was used as the control group. Patients were administered 0.1 mg of triptorelin daily from menstrual cycle day 2 (MC2) to the time of human chorionic gonadotropin (HCG) administration, and exogenous hMG (150–225 IU) was administered daily beginning on MC3. After 4–5 days of hMG administration, a transvaginal ultrasound examination and serum hormone level tests were performed and the dose of hMG was optimally adjusted based on the number and size of developing follicles. The final stage of oocyte maturation was induced with an intramuscular injection of hCG (Lizhu Pharmaceutical Trading Co., Zhuhai, China) after the dominant follicles had reached 18 mm in diameter. If a patient had more than three dominant follicles, 2,000 IU of hCG was administered, while for patients with no more than three dominant follicles, 5,000 IU of hCG was used for triggering. Transvaginal ultrasound-guided oocyte retrieval was conducted 34–36 h later.

#### PPOS protocol

A detailed description of the hMG + MPA procedure has been presented in our previous publication^17^. Briefly, ovarian stimulation was initiated for patients from MC3 onward, via the injection of 150–225 IU of hMG (Anhui Fengyuan Pharmaceutical Co. Ltd, Hefei, China) and MPA (4 mg/d or 10 mg/d)^17^. The initiating dose of 150 IU/day was used for patients with high AFC counts greater than 20 or slightly elevated basal FSH (7–10 IU/L), while 225 IU/day was used for all other patients. Prior contraception studies indicated that 10 mg MPA can be used to inhibit ovulation, while 5 mg MPA failed to inhibit ovulation^54^, it is likely that 10 mg MPA may be better to prevent premature LH surge in the milieu of controlled ovarian stimulation. In this trial, to explore the optimal MPA dose, we chose a dose of 4 mg and 10 mg MPA for further dose-effectiveness research. When three dominant follicles reached 18 mm in diameter, the final stage of oocyte maturation was cotriggered with SC injections of triptorelin (0.1 mg, Decapeptyl, Ferring Pharmaceuticals) and hCG (1,000 IU; Lizhu Pharmaceutical Trading Co.). Oocytes were retrieved via transvaginal aspiration 34–36 h after ovulation was triggered.

### IVF outcomes assessment

Patients underwent IVF treatment, which was performed according to the routine laboratory procedures on the day of oocyte retrieval as previously described^55, 56^. Briefly, Flushing Medium (Origio Medical Company, Denmark) was used for oocyte retrieval, and Human Tubal Fluid (HTF; Irvine Scientific, CA, USA) with 10% Serum Substitute Supplements (SSS; Irvine Scientific, CA, USA) was used as the oocyte collection and insemination medium. The embryos were cultured in 10%-SSS supplemented Continuous Single Culture medium (CSC; Irvine Scientific, CA, USA).

The day-3 embryos from each IVF treatment cycle were examined for the number and regularity of blastomeres and the degree of embryonic fragmentation according to Cummins’ criteria^57^. Grade I and II embryos, which were regarded as top-quality, were cryopreserved; Grade III and IV embryos were placed in extended culture until they reached the blastocyst stage, and only morphologically good blastocysts were frozen on day 5 or day 6. The vitrification procedure for freezing cleavage-stage embryos and blastocysts was performed using the Cryotopcarrier system (Kitazato Biopharma Co.) as previously reported^58^. For vitrification, a cryotop carrier system was utilized, with a mixture of 15% (v/v) ethylene glycol (EG), 15% (v/v) dimethyl sulfoxide (DMSO), and 0.5 M sucrose as the cryoprotectant solution. For thawing, solutions of 1, 0.5, and 0 M sucrose were used for the step-by-step cryoprotectant dilution.

### Endometrial Preparation and Frozen Embryo Transfer

Endometrial preparation for every transfer cycle was performed in a natural cycle, a mild stimulation cycle or a hormone replacement therapy as described in our previous article^19, 21^. Our method of embryo and endometrium synchronizationin FET was as follows: for natural FET cycles, follicular growth was monitored by serum hormones and ultrasound from cycle day 10. When endometrial thickness was >8 mm and the diameter of the dominant follicle was >16 mm, with E2 > 150 pg/mL and P < 1.0 ng/mL, one of two procedures was performed depending upon the LH value. If LH was <20 IU/L, we injected hCG 10,000 IU at night (21:00) to trigger ovulation and arranged the transfer of 3-day-oldembryos for 5 days later. If the LH value was >20 IU/L, hCG 10,000 IU was administrated the same afternoon and the transfer time was conducted 4 days later. The transfer of blastocysts was arranged for the 6 or 7 days later depending on serum hormones and ultrasound results. Dydrogesterone (Duphaston; Abbott Biologicals B.V., America) 40 mg/day was used for luteal phase support beginning on the 3rd day after hCG injection.

For some patients with irregular menstrual cycles after ovarian stimulation who originally had the regular menstrual cycles before the first ovarian stimulation, we used letrozole 2.5–5 mg administered from cycle day 3 to 7 to stimulate mono follicular growth. Follicle growth was monitored from cycle day 10. In some women, hMG at a dose of 75 IU/d was used to stimulate follicular and endometrial lining growth. Administration of 10,000 IU of hCG and the timing of FET were performed according to the above criteria.

For patients with thin endometria during either natural cycles or stimulation cycles, hormone therapy was recommended for endometrial preparation, oral E2 (ethinylestradiol; Shanghai Xinyi Pharma) 75 mg/day was used from cycle day 3 onwards. Once the endometrial thickness was >8 mm, Femoston (Solvay Pharmaceuticals B.V.) 8 mg/day was started. Embryo transfer was determined on the third day after Femoston administration.

In all FET cycles, one or two thawed embryos were transferred according to the patient’s intention. Once pregnancy was achieved, the exogenous estrogen and progesterone supplement were continued until 8–10 weeks of gestation.

### Statistical Analysis

All statistical analyses were performed with the use of the Statistical Package for the Social Sciences software (SPSS, version 17.0, SPSS Inc., Chicago, IL, USA) or GraphPad Prism 5 (GraphPad software Inc., La Jolla, CA 92037 USA). The outcome measures for this study included the number of oocytes retrieved, the number of mature oocytes, the number of fertilized oocytes, the number of viable embryos, number of top-quality embryos, and pregnancy and live birth outcomes from FETs. The implantation rate was defined as the number of gestational sacs divided by the number of embryos transferred. Biochemical pregnancy rate was defined as the proportion of patients with positive β-human chorionic gonadotrophin (β-HCG) in blood or urine. Clinical pregnancy was defined as the presence of a gestational sac with or without fetal heart activity under ultrasound examination 7 weeks after FET. The early miscarriage rate was defined as the proportion of patients with spontaneous pregnancy termination before the gestational age of 12 weeks. The live-birth rate was defined as the proportion of patients with live birth among all transfered cycles. In the study, the data were presented as the mean ± SEM. The one-way ANOVA analysis was used for the comparison of data in three groups if the normality assumption was true; otherwise, the nonparametric test was applied. Comparison between two groups, the number data were analyzed using Student’s *t*-tests if the normality assumption was true; otherwise, the Mann-Whitney *U*-test was applied. Proportions were compared using the chi-square test when appropriate. Logistic regression was performed on the clinical outcomes between three ovary stimulation groups separately in two BMIs groups. The differences were considered statistically significant when the *P*-value was less than 0.05.
